# Dynamic Neural Patterns of Human Emotions in Virtual Reality: Insights from EEG Microstate Analysis

**DOI:** 10.3390/brainsci14020113

**Published:** 2024-01-23

**Authors:** Yicai Bai, Minchang Yu, Yingjie Li

**Affiliations:** 1 School of Life Sciences, Shanghai University, Shanghai 200444, China; yicaibai@shu.edu.cn; 2 School of Communication and Information Engineering, Shanghai University, Shanghai 200444, China; minchang_yu@shu.edu.cn; 3 College of International Education, Shanghai University, Shanghai 200444, China

**Keywords:** EEG, microstate, virtual reality, emotions, dynamic features, transition pairs

## Abstract

Emotions play a crucial role in human life and affect mental health. Understanding the neural patterns associated with emotions is essential. Previous studies carried out some exploration of the neural features of emotions, but most have designed experiments in two-dimensional (2D) environments, which differs from real-life scenarios. To create a more real environment, this study investigated emotion-related brain activity using electroencephalography (EEG) microstate analysis in a virtual reality (VR) environment. We recruited 42 healthy volunteers to participate in our study. We explored the dynamic features of different emotions, and four characteristic microstates were analyzed. In the alpha band, microstate A exhibited a higher occurrence in both negative and positive emotions than in neutral emotions. Microstate C exhibited a prolonged duration of negative emotions compared to positive emotions, and a higher occurrence was observed in both microstates C and D during positive emotions. Notably, a unique transition pair was observed between microstates B and C during positive emotions, whereas a unique transition pair was observed between microstates A and D during negative emotions. This study emphasizes the potential of integrating virtual reality (VR) and EEG to facilitate experimental design. Furthermore, this study enhances our comprehension of neural activities during various emotional states.

## 1. Introduction

Emotions constitute a fundamental aspect of the human experience and pivotally influence our daily lives [1]. These emotional states are intricately intertwined with neural activity, necessitating a comprehensive understanding of the distinct neural patterns associated with various emotions [2]. In investigating neural activities, electroencephalography (EEG) is an effective tool. Previous EEG studies have employed neuroscience approaches to explore the neural features of emotions, such as spectral power and inter-hemispheric power asymmetry. For example, different emotions exhibited power asymmetry of the alpha band in the midfrontal and anterior temporal brain regions [3]. To date, most related experiments have traditionally been conducted in 2D environments, utilizing monitors to display experimental materials.

However, these settings deviate significantly from real-life situations and lack depth-of-field information and interactive sensations. Studies have indicated disparities in the neural mechanisms between these 2D artificial conditions and real-life situations. For instance, research has revealed enhanced functional connectivity in frontal and parietal brain regions in less immersive environments [4]. Therefore, the conclusions drawn from experiments utilizing 2D stimuli may not be directly applicable to the intricate real world. Virtual reality (VR), with its heightened fidelity to reality compared to conventional 2D paradigms, offers a unique perspective. However, despite the availability of publicly accessible EEG datasets on emotions, the majority are based on 2D videos sourced from movie clips, such as the famous DEAP and SEED EEG databases [5,6]. One database utilized only nine EEG channels to collect EEG in a VR environment. This study designed an emotion recognition system based on EEG spectral and connectivity analyses [7]. Another study classified the four emotions in the VREEG database (DER-VREEG), obtaining 85.01% classification accuracy [8]. We established a VR-EEG database, known as VREED, in 2021 [9]. Using VREED data, our investigations also demonstrated increased global efficiency in the frontal–occipital network in VR environments compared to 2D stimulation [10]. Few researchers have utilized these databases and most studies involving VR and EEG in the context of emotion have primarily concentrated on emotion categorization utilizing machine learning. However, less research investigated the neural activity during emotion processing.

An essential consideration in emotion experiments is the exploration of dynamic neural features. While some studies have concentrated solely on capturing static neural aspects, such as the classic left–right power asymmetry in the frontal lobe, dynamic features have often been overlooked. Emotions are inherently dynamic, and the neural activity is far from static. Moreover, the neural activity is not confined to isolated brain regions. For example, the experience of disgust is intricately connected to the insula and ventral prefrontal cortex [11]. Other research has revealed that heightened arousal levels are linked to a more star-shaped configuration in the minimum spanning tree theory [12]. Given the complexity of emotions, multiple brain regions are involved, each of which is characterized by unique dynamic features. In light of these complexities, our study aimed to explore both dynamic and whole-brain activity within emotion processing.

EEG microstates are powerful tools for addressing the research questions. These microstates refer to the scalp potential distributions exhibiting quasi-stable landscapes that maintain stability for approximately 100 ms. Microstate can shift from one state to another [13,14]. Notably, EEG microstates encapsulate spatial information and enable the dynamic observation of brain activity at millisecond intervals [15]. Because of the potential of microstates, a few studies have used them to investigate emotions. For instance, Prete et al. investigated the cerebral correlates of emotion processing [16]. Chen et al. proposed a k-mer-based feature extraction method for extracting features from emotional EEG data, achieving better EEG-based emotion recognition [17]. Hu et al. utilized correlation analysis to explore the relationship between microstate features, valence, and arousal. They found a positive association between the valence level and microstate D activities, as well as a negative association between the arousal level and microstate C activities [18]. Liu et al. investigated microstate features across nine discrete emotions, providing evidence that the multivariate differences in microstate features among the emotional conditions support the existence of distinct neural representations for discrete emotions [19].

Four commonly used microstates (A, B, C, and D) have been extensively studied in the field of neural science. These correspond to distinct brain networks, with insights derived from fMRI studies [20]. Microstate A exhibited positive and negative voltage distributions in the right prefrontal and left occipital lobes, which aligned with the fMRI auditory network. Microstate B, characterized by positive and negative voltage distributions in the left prefrontal and right occipital lobes, corresponds to the fMRI visual network. Microstate C, which displayed an anterior–posterior orientation, aligned with the fMRI salience network. Microstate D, with extreme values in the middle of the parietal lobe, corresponded to the fMRI dorsal attention network (DAN). The distributions of the four microstates exhibit remarkable consistency across the populations and states. Microstate features were assessed based on temporal properties, including globally explained variance (GEV), duration, occurrence, coverage, and transition probability. The GEV represents the proportion of variance explained by specific components in the EEG signal, typically exceeding 70% [15,21]. Duration represents the average persistence time of a microstate and reflects synchronized neuronal activity [14]. Occurrence denotes the frequency of appearances per second. Coverage indicates the proportion of the total time spent in a specific microstate, and transition probability reflects the likelihood of shifting from one microstate to another. Transition probabilities are crucial for investigating dynamic features and offering insights into the activation appearances of different brain networks. Microstate analysis facilitates the dynamic exploration of the whole brain.

Whether different emotions manifest distinct EEG microstate temporal dynamics in a VR environment remains unclear. This uncertainty arises from the fact that traditional EEG emotion studies did not incorporate VR technology. However, in contrast to those studies, this research takes a more realistic approach by utilizing VR technology. We hypothesized that various emotions would entail diverse whole-brain dynamic features in a VR setting.

We conducted a within-subject study employing a video-watching paradigm. By using scene clips presented on a VR device, we induced diverse emotions in the participants. Subsequently, we meticulously analyzed EEG features using microstate analysis. We clustered the four classic microstates, extracted their parameters, conducted a comprehensive analysis, and provided detailed explanations of pair features. Ultimately, we identified notable differences in both the duration and occurrence within the alpha band. Furthermore, specific transition pairs associated with different emotions were identified. In particular, in negative emotions, we observed a distinctive transition pair between microstates A and D in the alpha band. Conversely, for positive emotions, microstates B and C show unique transition pairs in the alpha band. These findings have the potential to serve as discriminative features for distinguishing between various emotions. Our results significantly contribute to a deeper understanding of how the brain processes emotions using an EEG microstate analysis.

## 2. Materials and Methods

### 2.1. Scene Selection and Participants

**Figure 1:**
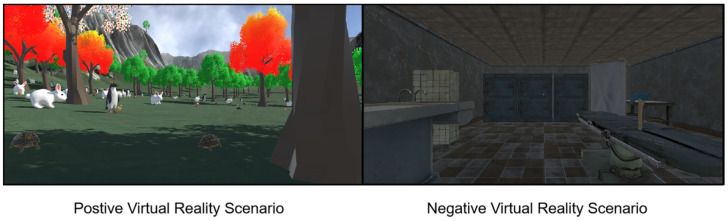
Positive and negative scene clips in the experiment.

Initially, we carefully selected four positive and four negative one-minute scenes for our study, as shown in Figure 1. To validate the suitability of our scene choices, we conducted a pre-experiment where twenty individuals evaluated the emotional content of the VR scenes. Notably, the participants could easily distinguish between positive and negative scenes. Subsequently, the required group size was determined using the G*Power program [22]. For our repeated measures analysis of variance (ANOVA), we set α = 0.05, effect size = 0.25, and a desired power (1 − β) of 0.8, obtaining a group size of 28. Similarly, for the paired *t*-test, we employed a two-sided test, set α = 0.05, effect size = 0.5, and aimed for a power (1 − β) of 0.8, resulting in a group size of 34. Ultimately, 42 healthy volunteers, with a sex distribution of 21 males and 21 females, with a mean age of 22.8 years and a standard deviation (SD) of 1.81 years, participated in our study. The number of participants exceeded the theoretical requirements. All participants were affiliated with Shanghai University and provided informed consent before the experiment. The participants received compensation for their participation. The experimental protocol was approved by the Shanghai Ethics Committee for Clinical Research and adhered to the principles outlined in the Declaration of Helsinki.

### 2.2. Emotion Induction and EEG Recording

**Figure 2:**
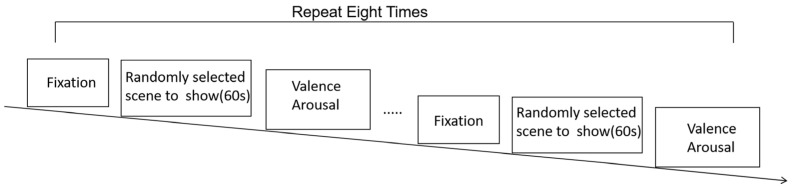
Experimental procedure.

During the experiment, the participants sat comfortably, and scenes were presented using an HTC VR headset. Each scene was accompanied by background music, and the participants wore earphones for an immersive audiovisual experience. Eight unique scenes were randomly presented, and this process was repeated twice. Participants viewed each scene for one minute and then assessed their emotions in terms of both valence and arousal using a nine-point SAM scale [23]. Concurrently, behavioral data were collected. The experimental procedure is illustrated in Figure 2.

To confirm that the scenes elicited distinct emotions, a one-sided paired *t*-test was conducted on mean valence for the positive and negative clips. The results revealed a significant difference (t = 24.0, *p* < 0.001, effect size = 12.0); the mean values and standard deviations are listed in Table 1. This statistical analysis confirmed that positive and negative scenes effectively induced different emotional responses in participants.

**Figure 3:**
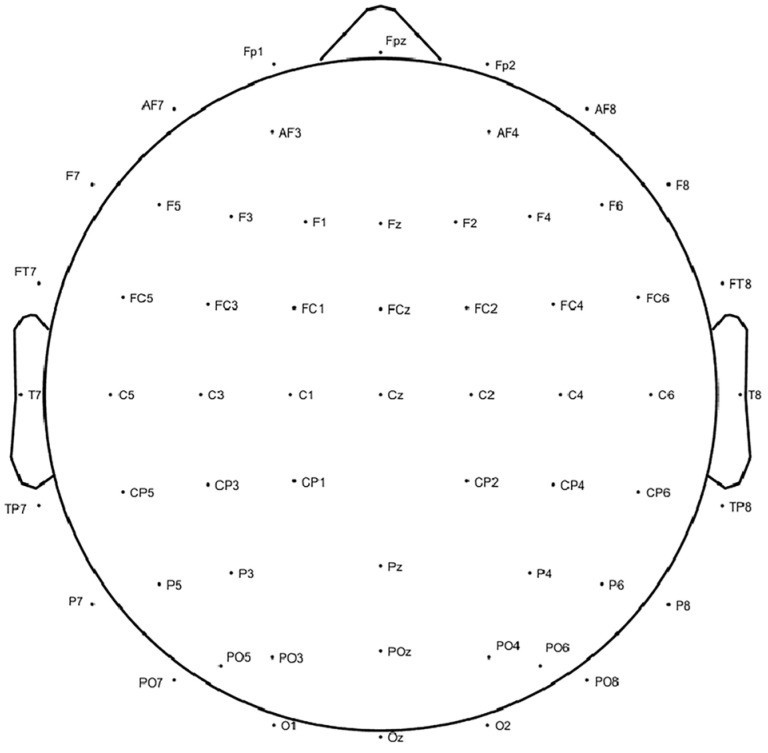
Electrode placement according to the 10–20 system.

Simultaneously, EEG signals were recorded, while the participants viewed the VR scenes using a 64-channel wireless EEG system provided by Neuracle Technology in Changzhou, China. Electrode placement followed the international 10–20 system, and data were sampled at a rate of 1000 Hz, with electrode impedance maintained below 5 kΩ. The number and placement of electrodes are depicted in Figure 3. Resting-state EEG signals were recorded before the participants engaged in the scenes.

### 2.3. EEG Pre-Processing

The EEG data were preprocessed using MATLAB version R2018b in conjunction with the EEGLAB toolbox version 14.1.1 [24]. Initially, we applied bandpass filtering to the data within the frequency range of 3–80 Hz and employed a notch filter to eliminate 50 Hz power frequency interference. Subsequently, electrodes not utilized in the experiment, such as ECG, HEOR, HEOL, VEOU, and VEOL, were excluded, and problematic channels were interpolated. Independent component analysis (ICA) or principal component analysis (PCA) was employed to eliminate artifacts originating from eye movements and muscle activity. Following artifact removal, the data were segmented into four-second intervals. Segments displaying significant noise or artifacts were excluded from further analysis. This includes segments with amplitudes greater than 100 μV and those exhibiting noticeable artifacts. Finally, average referencing was conducted, and the baseline was removed. The data were then divided into four frequency bands: theta (4–8 Hz), alpha (8–13 Hz), beta (13–30 Hz), and gamma (30–49 Hz) by using the pop_eegfiltnew function of EEGLAB (FIR band-pass filter of order 33000).

### 2.4. EEG Microstate Analysis

Microstate analysis was performed using the EEGLAB plugin (Microstates in EEGLAB, available at Thomaskoenig.ch, accessed on 18 November 2022) in MATLAB version R2021b. We focused on the initial 2-s segment of the EEG data following the presentation of the VR scenes. The microstate analysis procedure included template selection, back-fitting, and feature extraction for each trial. Previous studies have shown that microstates tend to remain stable when the global field power (GFP) reaches its maximum value, which indicates the highest topographic signal-to-noise ratio. They easily transition to another state when GFP reaches its minimum value [25]. GFP can be computed using Equation (1), where N represents the number of electrodes, and U represents the potential [12]. We employed only the data at these extreme values for subsequent clustering analysis without considering the polarity of the EEG signal.

(1) $$GFP=\sqrt{\frac{1}{N}\sum_{n=1}^{N}{\left(U_{n}(t)-\bar{U(t)}\right)}^{2}}$$

Taking into account the interpretability of the clustering algorithm, we applied the atomized-agglomerate hierarchical clustering (AAHC) algorithm to calculate the microstates within the theta (4–8 Hz), alpha (8–13 Hz), beta (13–30 Hz), and gamma (30–49 Hz) frequency bands. The AAHC operates as a bottom-up approach, initially treating each sample as a cluster and subsequently merging clusters with high similarity based on the global explained variance (GEV) value. This process iteratively reduces the number of states until the desired number of microstates is attained [21]. 

Emotions were categorized as positive, neutral, or negative based on valence ratings. To ensure data balance, valence ratings of 1, 2, and 3 were classified as negative emotions; valence ratings of 4 and 5 as neutral emotions; and valence ratings of 6, 7, 8, and 9 as positive emotions. Two participants did not report experiencing neutral emotions and one participant did not report experiencing positive emotions during the experiment. Consequently, these three individuals were excluded from the data analysis. The final dataset comprised information obtained from 39 participants, with a sex distribution of 20 males and 19 females. The participants had a mean age of 22.9 years, with a standard deviation (SD) of 1.73 years.

Four microstates were selected for microstate clustering. Initially, microstates were computed for each trial and averaged to create emotion-specific maps (negative, neutral, and positive). These emotion-specific maps for microstates were averaged to generate a global template. The emotion-specific templates were back-fitted using the global template as a reference. Finally, the microstate map of each trial was sorted based on the emotion-specific template [26]. Following these steps, the microstate parameters for each trial were obtained, resulting in 24 microstate features comprising 4 duration measures, 4 occurrence measures, 4 coverage measures, and 12 transition probabilities.

### 2.5. Statistics Analysis

After obtaining the EEG features, the data were exported to jamovi for statistical analysis. Jamovi 2.3.26 software was used to assess the differences among the various emotions. To mitigate variations stemming from different scene clips, we computed the average microstate features for each participant based on the emotion categories. A significance threshold of *p* < 0.05 was employed to denote statistically significant differences, which are highlighted in bold in the tables presented below. 

Repeated-measures analysis of variance (ANOVA) was employed to assess the differences in duration, occurrence, and coverage of different emotions. Normality tests were conducted, and based on the results, one-way repeated measures ANOVA or nonparametric Friedman tests were applied. If they did not match normality, we used the Friedman nonparametric repeated measures ANOVA test to assess differences among groups. If they matched normality, we used a one-way repeated measures ANOVA. Then, Mauchly’s test of sphericity was conducted, and sphericity corrections were performed where necessary. If the Greenhouse–Geisser value exceeded 0.75, we performed the Huynh–Feldt correction. If the Greenhouse–Geisser value was less than 0.75, we performed Greenhouse–Geisser correction [27]. Post hoc tests using the Bonferroni correction were conducted to examine the differences between the groups when the repeated measures ANOVA results indicated significant differences.

Transition probabilities were scrutinized using two-sided paired *t*-tests. Normality tests were conducted, and Wilcoxon tests were used for non-normally distributed data [27]. The neutral emotion was used as the reference, and paired *t*-tests were conducted for pairs such as neutral versus negative and neutral versus positive. Variations in the transition probability parameters provided insight into the dynamic transition tendencies of the microstate across different emotions.

## 3. Results

### 3.1. Cluster Evaluation

**Figure 4:**
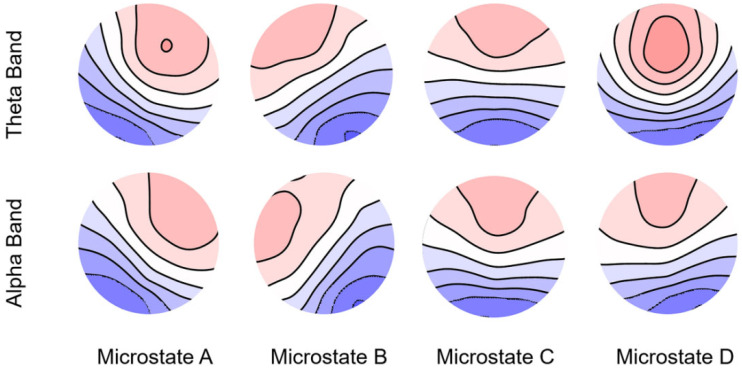
Spatial configuration of the four microstate classes in the theta and alpha bands.

The average GEV in the beta and gamma bands was <70%. Therefore, we conducted our analysis specifically for the theta and alpha bands. In the theta band, the average GEV was 76.30% (SD = 6.87%), 77.53% (SD = 7.10%), and 77.10% (SD = 7.40%) for negative, neutral, and positive emotions, respectively. The average GEVs in the alpha band were 78.81% (SD = 7.78%), 76.59% (SD = 8.00%), and 74.98% (SD = 7.97%) for negative, neutral, and positive emotions, respectively. The microstates corresponding to these results are shown in Figure 4.

### 3.2. Microstate Duration, Occurrence, and Coverage

No differences were observed in the theta band. Conversely, for the alpha band, differences were identified in duration and occurrence. Repeated measures ANOVA results showed statistical significance for the duration of microstate C (F(76) = 3.89, *p* = 0.025, η^2^ = 0.052). Regarding occurrence, statistically significant differences were observed in microstates A (F(76) = 6.42, *p* = 0.003, η^2^ = 0.072), C (F(76) = 3.78, *p* = 0.027, η^2^ = 0.064), and D (F(76) = 4.53, *p* = 0.014, η^2^ = 0.057). No statistically significant differences were found in the coverage.

**Figure 5:**
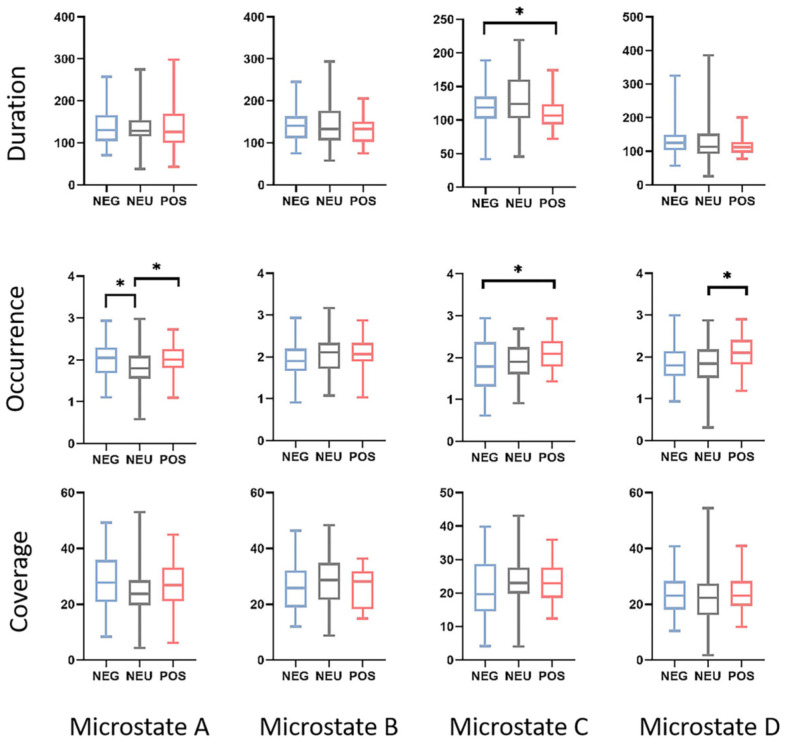
Within-factor differences in microstate duration, occurrence, and coverage using box plots and an ANOVA. In the figure, * indicates *p* < 0.05. The ends of the box plots represent the maximum and minimum values. The labels NEG, NEU, and POS correspond to the negative, neutral, and positive emotions, respectively. Detailed statistical information is presented in Table 2.

Post hoc tests with Bonferroni correction revealed specific differences between pairs of groups. Positive emotions exhibited a shorter duration in microstate C than neutral emotions (*p* = 0.034). In terms of occurrence, neutral emotions had a lower occurrence in microstate A than both positive (*p* = 0.033) and negative emotions (*p* = 0.014). Additionally, positive emotions showed a higher occurrence in microstate C than in negative emotions (*p* = 0.020), and positive emotions exhibited a higher occurrence in microstate D than in neutral emotions (*p* = 0.026). Figure 5 visually shows the statistical results.

**Table 2:** Analysis of microstate duration, occurrence, and coverage for microstates A–D between negative, neutral, and positive emotions.

	Class	NEG	NEU	POS	ANOVAs	Post Hoc (*p*-Value)		
						NEG- NEU	NEG- POS	NEU- POS
Duration/ms	A	138 ± 48.5	134 ± 47.5	139 ± 50.2	χ^2^(2) = 0.513, *p* = 0.975 ^a^	-	-	-
	B	140 ± 38.9	144 ± 50.1	130 ± 30.7	F(55.55) = 1.43, *p* = 0.246 ^b^ η^2^ = 0.021	-	-	-
	C	119 ± 32.1	130 ± 39.2	111 ± 24.4	F(64.7) = 3.89, ***p* = 0.032 ^b^** η^2^ = 0.052	0.481	0.447	**0.034**
	D	130 ± 43.9	129 ± 61.5	116 ± 28.5	χ^2^(2) = 4.97, *p* = 0.083 ^a^	-	-	-
Occurrence	A	2.08 ± 0.414	1.81 ± 0.522	2.02 ± 0.318	F(76) = 6.42, ***p* = 0.003 ^c^** η^2^ = 0.072	**0.014**	1.000	**0.033**
	B	1.93 ± 0.459	2.01 ± 0.460	2.08 ± 0.328	F(76) = 1.59, *p* = 0.210 ^c^ η^2^ = 0.020	-	-	-
	C	1.81 ± 0.516	1.89 ± 0.455	2.09 ± 0.380	F(76) = 3.78, ***p* = 0.027 ^c^** η^2^ = 0.064	1.000	**0.020**	0.114
	D	1.86 ± 0.464	1.83 ± 0.517	2.08 ± 0.418	F(76) = 4.53, ***p* = 0.014 ^c^** η^2^ = 0.057	1.000	**0.056**	**0.026**
Coverage/%	A	27.9 ± 10.0	24.7 ± 10.9	27.0 ± 8.38	χ^2^(2) = 1.28, *p* = 0.527 ^a^	-	-	-
	B	26.4 ± 8.82	28.4 ± 9.98	26.1 ± 7.07	χ^2^(2) = 1.33, *p* = 0.515 ^a^	-	-	-
	C	21.7 ± 9.20	23.5 ± 7.32	23.2 ± 6.01	F(65.53) = 0.738, *p* = 0.463 ^b^ η^2^ = 0.011	-	-	-
	D	24.0 ± 7.79	23.4 ± 10.7	23.8 ± 6.83	χ^2^(2) = 0.974, *p* = 0.614 ^a^	-	-	-

Values are presented as mean ± standard deviation. ^a^ Friedman test; ^b^ one-way repeated measures ANOVA with sphericity corrections; ^c^ one-way repeated measures ANOVA; *p*-values less than 0.05 are shown in bold type. NEG, negative emotion; NEU, neutral emotion; POS, positive emotion.

### 3.3. Analysis of Transition Probability in Microstates

**Figure 6:**
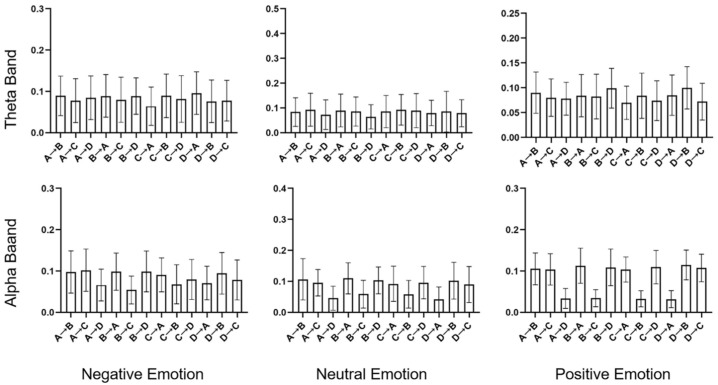
Transition probabilities for negative, neutral, and positive emotions in the theta and alpha bands. The histogram represents the mean and standard deviation values.

**Figure 7:**
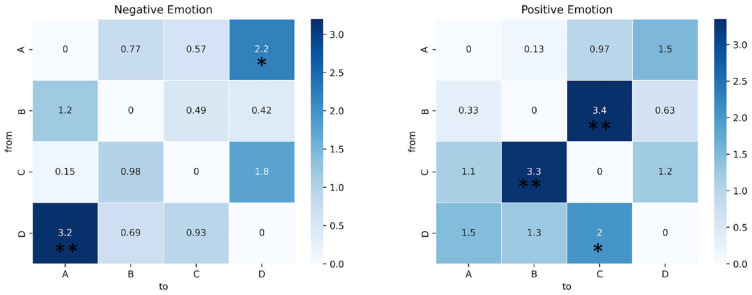
Paired t-test results of transition probability in the alpha band. The heat map shows the *t* statistics. * indicates *p* < 0.05, ** indicates *p* < 0.01. See Table 3 for more detailed statistical information.

For the analysis of the transition probabilities, paired *t*-tests were conducted, and the results are presented in Table 3. Figure 6 shows the transition probabilities for negative, neutral, and positive emotions in both the theta and alpha bands. Significant differences were observed for both bands. In particular, in the theta band, both negative and positive emotions exhibited higher transition probabilities from B to D compared to neutral emotion (*p* = 0.037, effect size = −0.346, and *p* = 0.001, effect size = −0.562, respectively). More significant differences were observed in the alpha bands. Negative emotions displayed higher transition probabilities from A to D (*p* = 0.037, effect size = −0.354) and from D to A (*p* = 0.037, effect size = −0.513) than neutral emotions. In contrast, positive emotions showed lower transition probabilities from B to C (*p* = 0.037, effect size = 0.536) and from C to B (*p* = 0.037, effect size = 0.530) than neutral emotions. And it had a higher transition probability from D to C (*p* = 0.037, effect size = −0.328). The results of the paired *t*-test for the alpha band are shown in Figure 7.

## 4. Discussion

We conducted an in-depth exploration of EEG microstate features associated with various emotions in a VR environment. By incorporating VR devices, we aimed to enhance the validity of our experimental setup and employed EEG microstate analysis to uncover the dynamic spatial-temporal features. Our results shed light on the variations in microstate duration, occurrence, and transition probabilities across different emotional states, with a specific focus on distinctive transition pairs within the alpha band. Notably, this study is the first to integrate microstate analysis with VR technology to research emotions, providing novel insights into the neural underpinnings of emotion processing.

### 4.1. Microstate Features Altered by Emotions

Our study examined different microstate features that are closely linked to emotions. Although no differences were observed in theta bands, differences were detected in alpha bands. The alpha band, which is associated with attention, yielded more significant findings, consistent with prior research [28,29]. Additionally, studies have also indicated that the generation of microstates is primarily associated with the alpha frequency band [30]. Repeated-measures ANOVA revealed significant differences in microstates A, C, and D. Microstate A is linked to major depressive disorders [31]. For positive and negative emotions, a greater occurrence of microstate A suggests that it is engaged in emotion processing. A previous study also found a difference in microstate B levels, which was explained by video stimulation [32]. Microstate B corresponds to the visual network, which includes the retinotopic occipital cortex and temporal-occipital regions [33,34]. In our study, we did not find any differences, possibly because we averaged the results from different scenes. Microstates C and D, which are associated with the high-level processing of emotions, displayed variations in duration and occurrence. Previous studies have indicated that microstate C is linked to arousal and valence, respectively [35,36]. The longer duration of microstate C during negative conditions reflects slower temporal dynamics compared with positive emotions, which is consistent with the daily experience of reduced efficiency during negative emotional states. The occurrence of microstate C was lower for negative emotions, possibly because of its longer duration. Furthermore, our study found a higher occurrence of microstate D, which may indicate faster and more flexible temporal dynamics of positive emotions [37]. A previous study showed that valence level is positively correlated with the occurrence of microstate D [20]. This trend was consistent with our observations. However, this trend only shows positive and neutral emotions. This is possibly due to differences in the EEG signal frequency bands, clustering methods and algorithms, and the VR environment. Given that neural activity may differ in the real 3D world, our study reminds researchers of the need for an updated exploration of the presentation of experimental materials.

### 4.2. Unique Dynamic Transition in Different Emotions

**Figure 8:**
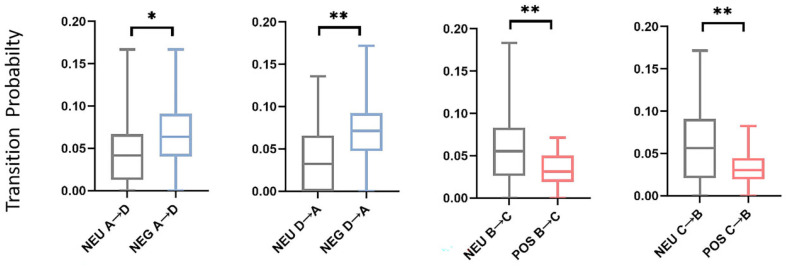
Dynamic microstate transition probability pairs. The ends of the box plot represent the maximum and minimum values. * indicate *p* < 0.05, ** indicate *p* < 0.01. NEG, negative emotion; NEU, neutral emotion; POS, positive emotion. See Table 3 for more detailed statistical information.

Non-random microstate transitions provide unique temporal dynamic features [38]. Specific transition pairs were identified for different emotions. Unique transition pairs were observed in the alpha band. The dynamic microstate transition pairs for the alpha band are shown in Figure 8. In negative emotions, the transitions from A to D and from D to A both have higher probabilities compared with neutral emotions; in positive emotions, transitions from B to C and from C to B both have lower probabilities compared with neutral emotions. These results show that negative emotions exhibited stronger connections between microstates A and D, while positive emotions displayed weaker connections between microstates B and C. Reflections on the brain network show that the connection between the auditory network and the dorsal attention network (DAN) increases during negative emotions, and the connection between the visual network and salience network decreases during positive emotions. In positive emotions, the transition from D to C also shows a difference. Positive emotions exhibit complex transitions, indicating active cognitive engagement in positive emotional states. Processing positive emotions may involve complex brain networks [35]. These unique transition pairs hold promise as potential features for distinguishing between emotional states.

### 4.3. Limitations and Future Work

This study has several limitations that merit consideration. Our simplified approach categorized emotions broadly based on valence. In contrast, some studies have delved into more discrete emotions, necessitating a larger variety of video stimuli to effectively induce such emotions [19]. Future studies should explore discrete emotions by using a wider array of stimuli. Additionally, there is currently no consensus on standardized microstate models. Different research studies employ different microstate models for identifying microstates, leading to difficulties in comparing results across studies. Future research is required to reach a consensus in this area.

Additionally, addressing emotion regulation through microstates and exploring strategies for emotional regulation via feedback mechanisms may be fruitful avenues for future research. Moreover, the evaluation of microstates in EEG data requires further supplementation. For example, Chen et al. proposed the dual-threshold-based atomize and agglomerate hierarchical clustering (DTAAHC) method to cluster the microstates, thus providing supplementary and optimized approaches to the existing research methods for EEG microstates and the classification of emotions [39]. New approaches could be explored in future studies. In addition, we found a lower global explained variance (GEV) at higher frequencies when considering the same number of microstates. However, this requires further investigation.

## 5. Conclusions

Our study employed EEG microstate analysis to investigate the whole-brain perspective and dynamic features of different emotions in a VR environment. In this within-subject experiment, we made an intriguing observation regarding the alpha band. In particular, we found that microstate A exhibited higher occurrence during the brain processing of emotions, indicating its involvement in emotion processing. Moreover, microstates C and D displayed faster dynamic features during positive emotions. Furthermore, we identified distinct transition patterns across emotions in the alpha band. In the context of negative emotions, we observed higher transition probabilities from microstate A to D and from D to A compared to neutral emotions. This suggests that there are stronger connections between the auditory and dorsal attention networks (DANs) during negative emotional processing. Conversely, during positive emotions, we found lower transition probabilities from microstates B to C and from C to B compared to neutral emotions, indicating weaker connections between the visual and salience. These findings provide valuable insights into the neural activities associated with emotions and present a novel exploration of this topic.

## Figures and Tables

**Table 1:** Mean valence values of positive and negative scenes.

	Emotion Condition	
	Negative	Positive
valence	3.25 (0.077)	6.45 (0.196)

**Table 3:** Results of paired *t*-tests for microstate transition probabilities between negative, neutral, and positive emotions.

Transition Probability	Emotion and Frequency Band			
	Negative (4–8 Hz)	Positive (4–8 Hz)	Negative (8–13 Hz)	Positive (8–13 Hz)
A-B	t = −0.528 *p* = 0.600 ^a^	w = 348 *p* = 0.566 ^b^	t = 0.771 *p* = 0.445 ^a^	t = 0.127 *p* = 0.899 ^a^
A-C	t = 1.14 *p* = 0.262 ^a^	w = 407 *p* = 0.602 ^b^	t = −0.568 *p* = 0.573 ^a^	t = −0.967 *p* = 0.339 ^a^
A-D	t = −0.909 *p* = 0.369 ^a^	w = 295 *p* = 0.189 ^b^	t = −2.21 *p* = 0.033 ^a^	t = 1.52 *p* = 0.136 ^a^
B-A	t = −0.529 *p* = 0.958 ^a^	t = 0.497 *p* = 0.622 ^a^	t = 1.19 *p* = 0.241 ^a^	t = −0.325 *p* = 0.747 ^a^
B-C	t = 0.452 *p* = 0.654 ^a^	t = 0.285 *p* = 0.777 ^a^	t = 0.487 *p* = 0.629 ^a^	t = 3.35 *p* = 0.002 ^a^
B-D	**t = −2.16 *p* = 0.037 ^a^**	**t = −3.51 *p* = 0.001 ^a^**	t = 0.419 *p* = 0.677 ^a^	t = −0.634 *p* = 0.530 ^a^
C-A	t = 1.79 *p* = 0.081 ^a^	t = 1.75 *p* = 0.088 ^a^	t = 0.151 *p* = 0.881 ^a^	t = −1.14 *p* = 0.263 ^a^
C-B	t = 0.299 *p* = 0.767 ^a^	t = 0.762 *p* = 0.451 ^a^	t = −0.981 *p* = 0.333 ^a^	t = 3.31 *p* = 0.002 ^a^
C-D	t = 0.663 *p* = 0.511 ^a^	t = 1.32 *p* = 0.193 ^a^	t = 1.76 *p* = 0.087 ^a^	t = −1.24 *p* = 0.221 ^a^
D-A	t = −1.40 *p* = 0.169 ^a^	t = −0.559 *p* = 0.597 ^a^	**t = −3.20 *p* = 0.003 ^a^**	t = 1.47 *p* = 0.149 ^a^
D-B	w = 414 *p* = 0.746 ^b^	w = 259 *p* = 0.068 ^b^	t = 0.690 *p* = 0.494 ^a^	t = −1.27 *p* = 0.213 ^a^
D-C	t = 0.109 *p* = 0.914 ^a^	t = 0.824 *p* = 0.415 ^a^	t = 0.925 *p* = 0.361 ^a^	**t = −2.05 *p* = 0.041 ^a^**

*p*-values less than 0.05 are shown in bold type. ^a^ paired *t*-test. ^b^ Wilcoxon test.

## Data Availability

The data presented in this study are available on request from the corresponding author. The data are not publicly available due to privacy restrictions.
